# Assessing ChatGPT’s Capability as a New Age Standardized Patient: Qualitative Study

**DOI:** 10.2196/63353

**Published:** 2025-05-20

**Authors:** Joseph Cross, Tarron Kayalackakom, Raymond E Robinson, Andrea Vaughans, Roopa Sebastian, Ricardo Hood, Courtney Lewis, Sumanth Devaraju, Prasanna Honnavar, Sheetal Naik, Jillwin Joseph, Nikhilesh Anand, Abdalla Mohammed, Asjah Johnson, Eliran Cohen, Teniola Adeniji, Aisling Nnenna Nnaji, Julia Elizabeth George

**Affiliations:** 1 Medical University of the Americas, PO Box 701, Charlestown, Saint Kitts and Nevis, 1 9788629500 ext 364; 2 Department of Education Enhancement, College of Medicine, American University of Antigua, St Johns, Antigua and Barbuda; 3 Department of Health Informatics, School of Professional Studies, Northwestern University, Evanston, IL, United States; 4 Department of Biochemistry, Cell Biology and Genetics, College of Medicine, American University of Antigua, Basseterre, Antigua and Barbuda; 5 Department of Medical Education, School of Medicine, University of Texas Rio Grande Valley, Edinburgh, TX, United States; 6 School of Medicine, Xavier University, Orangestad, Aruba

**Keywords:** medical education, standardized patient, AI, ChatGPT, virtual patient, assessment, standardized patients, LLM, effectiveness, medical school, qualitative, flexibility, diagnostic

## Abstract

**Background:**

Standardized patients (SPs) have been crucial in medical education, offering realistic patient interactions to students. Despite their benefits, SP training is resource-intensive and access can be limited. Advances in artificial intelligence (AI), particularly with large language models such as ChatGPT, present new opportunities for virtual SPs, potentially addressing these limitations.

**Objectives:**

This study aims to assess medical students’ perceptions and experiences of using ChatGPT as an SP and to evaluate ChatGPT’s effectiveness in performing as a virtual SP in a medical school setting.

**Methods:**

This qualitative study, approved by the American University of Antigua Institutional Review Board, involved 9 students (5 females and 4 males, aged 22‐48 years) from the American University of Antigua College of Medicine. Students were observed during a live role-play, interacting with ChatGPT as an SP using a predetermined prompt. A structured 15-question survey was administered before and after the interaction. Thematic analysis was conducted on the transcribed and coded responses, with inductive category formation.

**Results:**

Thematic analysis identified key themes preinteraction including technology limitations (eg, prompt engineering difficulties), learning efficacy (eg, potential for personalized learning and reduced interview stress), verisimilitude (eg, absence of visual cues), and trust (eg, concerns about AI accuracy). Postinteraction, students noted improvements in prompt engineering, some alignment issues (eg, limited responses on sensitive topics), maintained learning efficacy (eg, convenience and repetition), and continued verisimilitude challenges (eg, lack of empathy and nonverbal cues). No significant trust issues were reported postinteraction. Despite some limitations, students found ChatGPT as a valuable supplement to traditional SPs, enhancing practice flexibility and diagnostic skills.

**Conclusions:**

ChatGPT can effectively augment traditional SPs in medical education, offering accessible, flexible practice opportunities. However, it cannot fully replace human SPs due to limitations in verisimilitude and prompt engineering challenges. Integrating prompt engineering into medical curricula and continuous advancements in AI are recommended to enhance the use of virtual SPs.

## Introduction

Standardized patients (SPs) have been a cornerstone of medical education since the 1960s, offering students an immersive, real-world experience in a controlled environment. Studies have demonstrated that SP programs are superior for teaching consultation skills compared with traditional methods, with medical students trained using SPs showing increased confidence and competency compared with those trained through other modalities [1,2].

While SPs provide valuable opportunities for students to practice diagnostic and interpersonal skills under standardized conditions, several inherent challenges exist. The resource-intensive nature of SP programs has been a persistent issue, with significant costs associated with recruitment, training, and maintenance of an SP bank [1,3]. Additionally, questions have emerged about SPs’ ability to adequately represent the nuances of real patient presentations.

These challenges are particularly pronounced in specific contexts. For instance, Caribbean medical schools face unique obstacles due to limited local health care infrastructure and varying access to clinical training resources. Many offshore institutions in countries such as Aruba and Antigua and Barbuda must rely on partnerships with local health care providers, often resulting in inconsistent access across student cohorts [4,5]. The COVID-19 pandemic exposed additional vulnerabilities in traditional SP programs. The discontinuation of the USMLE Step 2 Clinical Skills examination in 2022, for instance, highlighted the risks of relying solely on in-person SP encounters for assessment [5].

In the 21st century, virtual SPs have emerged. These are computer programs that simulate specific illnesses and respond to learner inputs [6]. They have become invaluable tools in both teaching and assessment. However, their development also requires significant resources, making it challenging for institutions without robust educational technology support departments [7].

As the field of artificial intelligence (AI) has advanced, the potential for its application in medical education has expanded. Large language models (LLMs), such as ChatGPT (OpenAI), have revolutionized natural language processing. These sophisticated neural networks, trained on vast amounts of web-based data, are adept at predicting subsequent words in a sequence [8]. ChatGPT, a chatbot based on the GPT-3.5 model, has an enormous 175 billion parameters and displays a remarkable capacity for understanding and reasoning, bordering on human-like proficiency [9]. Since its introduction in November 2022, sectors spanning from history to entertainment have rapidly adopted the LLM [10].

This advancement in AI has led to the development of virtual SP chatbots. A number of major educational material suppliers and specialized companies are offering chatbot SPs, based on LLMs capable of natural language interactions, for students to practice clinical skills. One example is Oscer, which can present more than 200 virtual patient conditions and boasts above 90% accuracy in symptomology [11]. Similarly, the University of Texas Medical Branch makes use of an AI agent termed Virti, which they use to conduct virtual Observed Structured Clinical Examinations with medical students [12]. Other publicly accessible sites offering virtual patients include Soma Lab [13] and Body Interact [14]. However, for this new generation of virtual patients there is again considerable time and resources required for the company or the institution to develop the program and train the LLM on specific datasets and student access can be limited by cost and locality [7].

The debut of ChatGPT sparked inquiries into its potential as an SP. Liu et. al [15] crafted 10 medical histories with ChatGPT, which were then vetted by experienced physicians. Their results highlighted ChatGPT’s promise in clinical education, although some responses came across as robotic [15]. Suarez et.al [16] gathered dental student’s feedback after interacting with an AI chatbot. The majority found the experience valuable, especially those who made a correct diagnosis. This underscores the potential of integrating AI into health sciences training [16].

Weidener and Fischer [17] emphasized the growing consensus on incorporating AI into medical education. Their study indicated the importance of both practical and technological skills for leveraging AI in medicine [17]. Similarly, Jowsey et. al [18] have recommended adoption of AI into medical education as a way of preparing future physicians for the reality of modern practice.

We were aware that SPs at our school, American University of Antigua (AUA), were in limited supply and had received feedback indicating that while SPs are effective, students would like greater access to them. In fact, some students had no access during their course, depending on their cohort.

One of our study’s aims was to assess medical students’ perceptions and experiences regarding the use of AI in medicine—specifically by examining their views before and after interacting with ChatGPT as an SP. A second aim was to evaluate whether ChatGPT can perform adequately as a virtual SP in a medical school setting. Guided by these aims, our investigation focused on the following research questions: (1) How do students perceive the effectiveness of ChatGPT compared with traditional SPs in medical training scenarios? (2) To what extent can ChatGPT function effectively as a virtual SP in medical education?

By addressing these questions, our study seeks to inform the potential integration of AI-driven virtual SPs into medical curricula, particularly in settings where access to traditional SPs is limited.

## Methods

### Ethical Considerations

This study was given expedited approval by the AUA Research committee (no. AUAIRBa23011). Eleven medical student volunteers enrolled in the MD course at AUA were recruited via a campus-wide email. Two participants were lost to follow-up, leaving a total of 9 participants. Students were 5 females and 4 males, aged 22-48 years, comprising students from both first and second years of the basic sciences course section of the MD program. Participants were explicitly informed that their involvement in the research was completely voluntary. They were also assured that their responses would remain confidential and anonymous, and all participants signed informed consent agreements. All data were anonymized and no compensation was provided to participants.

### Study Design

**Figure 1.**
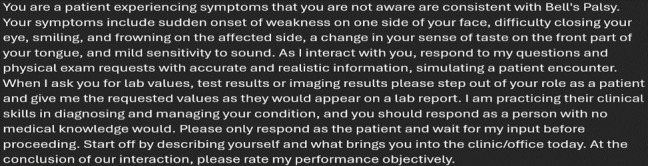
Prompt used in ChatGPT role-play.

Students were given access to ChatGPT version 4.0 accounts, the most recent available at the time of the study. Students were observed during a live role-play, in which a student inputted a predetermined prompt, provided by the study authors, into ChatGPT. The prompt directed the LLM to present as a patient with a neurological condition (Figure 1).

**Figure 2.**
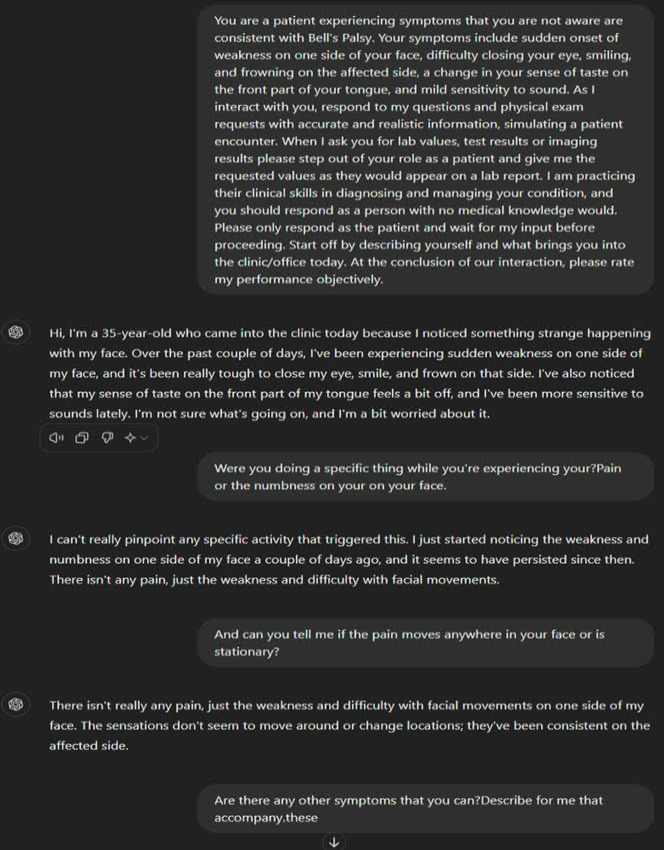
Screenshot example of ChatGPT standardized patient interaction.

The student, in the role of physician, then interviewed the ChatGPT and attempted to make a differential diagnosis (Figure 2).

### Prompt Development

The development of the prompt for ChatGPT’s simulated patient interaction underwent an iterative process prior to its use by students. This process involved a 6-member faculty team comprising both clinical and nonclinical faculty, ensuring a diverse range of perspectives and expertise. The faculty were tasked with using the prompt in simulated interactions with ChatGPT, assessing the following factors:

1. Consistency: ensuring the chatbot consistently adhered to the patient role and provided responses aligned with the illness script.
2. Accuracy: evaluating whether the responses were medically plausible and aligned with the provided case information.
3. Likelihood of misleading the SP: assessing whether the chatbot responses could inadvertently lead users to incorrect assumptions or conclusions.
4. Quality of output: reviewing the depth and appropriateness of responses to ensure a realistic and effective simulation experience.
5. Adherence to prompt instructions: verifying that ChatGPT’s responses followed the specific behavioral and informational instructions embedded in the prompt.

Faculty provided detailed feedback based on their observations, leading to refinements in the prompt. Suggestions included adjustments to phrasing, additional clarifications to the illness script, and enhancements to behavioral instructions to minimize the potential for ChatGPT to deviate from the assigned patient role. This iterative process was instrumental in optimizing the prompt’s effectiveness before deployment in the study.

### Rationale for Clinical Case Selection

Bell palsy was chosen as the clinical condition for the simulation due to its relevance to the material being taught at the time. This alignment ensured that the scenario was both clinically pertinent and integrated with the participants’ ongoing coursework in both basic sciences and clinical disciplines. The familiarity of the students with the foundational aspects of Bell palsy was intended to facilitate meaningful engagement with the simulated patient, allowing them to focus on the interaction and diagnostic questioning rather than struggling with unfamiliar content.

### Purpose of the Evaluation

It is important to note that the primary goal of this study was not to evaluate the students’ diagnostic accuracy. Instead, the focus was on assessing their perceptions of ChatGPT’s performance as a simulated patient. This distinction was critical to the study design, as it allowed for an emphasis on the usability, realism, and educational value of AI-driven SP interactions without conflating these aspects with the participants’ clinical competencies.

The role-play was conducted verbally, as a voice control extension added to the ChatGPT accounts allowed natural language conversation between the student and the LLM [19]. A structured questionnaire consisting of 15 open-ended questions was administered before and after interaction with ChatGPT in the role of an SP. Students were asked about specific elements of their interaction and interviews were conducted in person by faculty team members (Multimedia Appendix 1).

Participating students were introduced to the ethical considerations of using LLMs such as ChatGPT. This included training on the importance of deidentifying patient data, recognizing the limitations of AI, and understanding the potential biases inherent in AI responses, such as those related to gender or ethnicity. This ethical orientation aimed to ensure that students approached the interactions responsibly and with an awareness of the technology’s constraints.

### Thematic Analysis

The results of the students’ group work were recorded, transcribed, and coded by 3 different authors (JC, TK, and RER). Following discussions in regular meetings, findings were summarized, and a category system consisting of main and subcategories, according to Mayring’s [20] qualitative content analysis, was agreed upon. Selected text passages were used as quotations to illustrate each category. Inductive category formation, a qualitative research method used to analyze data by identifying patterns, themes, or categories that emerge directly from the data itself, without predefined hypotheses or coding frameworks, was used to analyze open-ended survey responses and interview transcripts.

To explore differences in prompt engineering techniques across academic levels, we asked students to describe how they approached questioning and refining their prompts during the postsession interviews. First-year students, who had less clinical exposure, were expected to rely more on general inquiry methods, while second-year students might leverage slightly more clinical insight. Recording these observations allowed us to compare prompt engineering strategies between these groups and understand how curriculum familiarity influenced interactions with the AI-driven simulated patient interactions.

## Results

A total of 9 students participated (5 females and 4 males, aged 22‐48 years) (Table 1). All students had had some prior experience with traditional SPs, with more senior students having had a greater number of encounters. This contextualizes their perceptions of ChatGPT as a supplement and provides a baseline for understanding the comparative effectiveness of the AI-based approach.

**Table 1.** Demographic data.

Characteristics	Participants (n=9), n
Age (years)^a^	
22‐30	4
31‐40	4
41+	1
Sex	
Male	4
Female	5
Semester	
1	0
2	7
3	1
4	1
5	0
Prior experience with SPs^b^	
Yes	9
No	0
Prior experience with AI^c^/ChatGPT	
Yes	6
No	3

a Mean age: 31.22 (SD 6.8) years

b SPs: standardized patients.

c AI: artificial intelligence.

The thematic analysis of student feedback prior to interaction with ChatGPT as an SP identified several key themes and subthemes (Table 2). Under the theme of technology limitations, students noted challenges with prompt engineering, such as difficulty in asking effective questions, because the AI could not role-play a physical examination. In terms of learning efficacy, students mentioned the potential for personalized learning materials, grammatical assistance, and the ability for repeated practice without the constraints of limited SP availability. Additionally, some students highlighted the potential for increased convenience, as they could practice as often and whenever they wanted. A potential reduction in SP interview stress was also seen as a benefit of increasing virtual practice. However, under the theme of verisimilitude (ie, the degree to which a simulation mirrors real-life scenarios, including the subtle behaviors and interactions that contribute to a convincing experience), students expressed concerns about the absence of visual cues and rapport, which are important in real patient interactions. Finally, trust issues were raised regarding the accuracy of the LLMs output.

**Table 2.** Thematic analysis of student feedback preinteraction with ChatGPT standardized patient.

Themes and subthemes	Representative quotations
Theme 1. Technology limitations	
Prompt engineering	“The challenges might be just asking the right questions, because it’s an AI, you can’t ask them to do physical examinations.”
Theme 2. Learning efficacy	
Personalized learning materials	“Triple checking work and not only getting the right answer, but getting explanations for the right answer and then why the wrong answer is wrong.”
Grammatical assistance	“It would be helpful because English is not my first language.”
Repetition	“There’s usually 10 medical students to one patient, and sometimes you’re fighting over each other to get the interview, so this allows us to get more repetitions.”
Depth of medical knowledge	“The sky’s the limit with regards to what we can practice.”
Interview stress or anxiety	“It will kind of be a bit more stress free because you know you’re talking to a computer rather than an actual patient.”
Convenience	“Be able to practice it as much as I want, as often as I want and any time I want.”
Theme 3. Verisimilitude	
Absence of visual cues	“You have to figure out ways to ask the question without the visual cues.”
Absence of rapport or empathy	“Building the communication and the relationship with your patient is important.”
Theme 4. Trust	
Inaccurate output	“One incident was in the small group activity, where it gave us the wrong answer.”

Following interaction with ChatGPT, the thematic analysis of student feedback revealed some changes in perceptions (Table 3). While technology limitations were still noted, students mentioned that they had learnt to improve the output from ChatGPT by tailoring prompts. They also reported alignment issues, such as ChatGPT not providing information on sensitive topics such as patient sexual history. Learning efficacy remained a significant theme, with students appreciating the convenience and repetition benefits. They found the ability to practice history taking without stress and receive feedback useful for skill development. However, verisimilitude issues persisted as a theme, with students noting the absence of visual and tonal cues, and the lack of rapport and empathy, all of which impacted the effectiveness of the patient interview and the ability to make a diagnosis. Some students experienced information overload, feeling that ChatGPT provided more information than a real patient would.

**Table 3.** Thematic analysis of student feedback postinteraction with ChatGPT standardized patient.

Themes and subthemes	Representative quotations
Theme 1. Technology limitations	
Prompt engineering	“You could put in the prompt that you want to tailor the responses you want to get back.”
Alignment	“When I asked like about sexual history, they were not able to give information.”
Theme 2. Learning efficacy	
Convenience	“Having ChatGPT to practice history whenever we want, I think that’s the improvement.”
Repetition	“You are able to have a lot more repetitions than you are in lab.”
Interview stress or anxiety	“Since it’s a computer, it’s not real. I had less anxiety.”
Feedback	“I can ask ‘hey, how did you think I did?’”
Skills development	“It highlighted the importance of on-the-spot thinking and memory recall in a medical scenario.”
Overall enhanced learning	“It’s going to make you sharper. You know, you’re probably going to be ahead of your peers, you’re going to be able to answer a patient in a better, more detailed manner. Give them a better treatment or care plan.”
Theme 3. Verisimilitude	
Absence of visual cues	“For the standardized patient you physically see them. You can see if they’re in pain, they don’t have to explain where they are in pain.”
Absence of tonal cues	“ChatGPT had the same tone, even if it was saying something sad.”
Absence of rapport or empathy	“It takes away the personal connection between the doctor and the patient.”
Information overload	“It felt like it was offering more information than a regular patient would.”

To provide broader context, we compared ChatGPT with some other virtual SP platforms or platforms that could provide this function (Table 4). The comparison highlights the unique strengths and weaknesses of ChatGPT identified in this study in comparison with other platforms, including Claude AI (another chatbot often ranking near the top of benchmarking tables), Body Interact, Oscer AI, and Soma Lab [13,14,21-23]. Both ChatGPT and Claude AI offer flexibility and unlimited practice but are limited by uncurated outputs and reliance on prompt engineering. Oscer AI and Som Lab provide curated clinical cases with tailored feedback, yet their visual representation and interactivity vary, with Soma Lab integrating natural conversational voice modes. Body Interact enhances verisimilitude through patient avatars and curated cases but lacks voice interaction. Cost structures range from free access for basic use to subscription-based models for advanced features.

**Table 4.** Comparison of various platforms able to function as standardized patients.

Platform	Technology limitations	Learning efficacy	Verisimilitude	Model cost
ChatGPT	Requires effective prompt engineering; uncurated outputs	Offers flexibility and unlimited practice	Limited visual and tonal cues; natural conversational voice mode	Free and subscription-based options
Claude AI	Requires effective prompt engineering; uncurated outputs	Offers flexibility and unlimited practice	Limited visual and tonal cues; limited voice interaction	Free and subscription-based options
Body Interact	Requires effective prompt engineering; curated clinical cases	Facilitates skill development through realistic scenarios	Patient avatars; lacks voice interaction	Subscription or licensing fees
Oscer AI	Requires effective prompt engineering; wide range of curated clinical cases	Focus on history-taking skills with limited versatility	Limited visual cues; voice interaction possible	Free. Subscription for full access
Soma Lab	Requires effective prompt engineering; wide range of curated clinical cases	Counseling-focused; supports repeated practice with tailored feedback	Static patient avatars; natural conversational voice mode	Variable costs based on usage and features

## Discussion

### Principal Findings

This study investigated the use of ChatGPT as an SP by qualitative analysis of students’ responses to a questionnaire, preinteraction and postinteraction, with ChatGPT performing the role of SP. In terms of diagnostic skill development, our conclusions were drawn from a combination of faculty observations and student self-report. Faculty members who observed the sessions noted that students demonstrated more structured reasoning and improved question formulation after repetitive practice with ChatGPT. In postsession interviews, students themselves expressed feeling more confident and organized in their clinical reasoning steps. This alignment between external observation and self-assessment suggests that the interaction with ChatGPT, although lacking nonverbal cues and certain realistic elements, still provides a valuable platform for honing diagnostic interviewing skills. Thematic analysis provided insights into student perceptions. Major themes identified were technology limitations, learning efficacy, and verisimilitude. Our results suggest that the current version of ChatGPT (ChatGPT version 4.0 at the time of this study) can function effectively as an augmentation to traditional SPs but cannot fully substitute for SPs. These results are broadly in line with those of other studies using LLMs in the role of SP [24-29].

The technological limitations of LLMs in the context of SP exercises were both anticipated and confirmed in our study. The subtheme of prompt engineering was particularly important. Students were made aware of the importance of correctly worded prompts before the exercise, and we found that the faculty-provided prompt, developed through a trial and error process, proved effective in this regard.

The significance of prompt engineering when using LLMs as virtual SPs, or in developing related materials, is also supported by other studies [28,30-33]. It has been suggested that prompt engineering could be incorporated into medical curricula through, for example, hands-on workshops, simulation-based learning, and courses on AI in health care [28,30-32].

The postinteraction interviews also revealed an additional subtheme of alignment. Alignment refers to the problem of ensuring that AI acts in accordance with human intentions and human values [34]. Students noted that the LLM did not provide a response when asked about a patient’s sexual history, a standard question in any medical consultation. Ensuring that ChatGPT does not output material which could be considered offensive under societal norms is a component of alignment [35]. However, our results demonstrate an “alignment tax,” in that the model becomes less useful due to constraints imposed by the alignment. The development of LLMs designed specifically for medical education may overcome this issue [36].

Learning efficacy was also a major theme identified in this study. Important subthemes in this category were repetition and convenience. Students noted the benefits of having access to ChatGPT for practice at any time or place and having virtually unlimited ability to repeat the exercises. As mentioned earlier, access to SPs is limited in many medical schools [15]. The ability to augment this shortfall with a virtual SP may be a positive option for many medical students and medical schools.

Interestingly, some students expressed that they experienced considerable anxiety as much as a day before they were scheduled to interact with an SP, although they were aware that the SP was not a real patient. The ability to practice with an LLM such as ChatGPT was seen as beneficial, because students could develop questioning techniques to a point where even during the session with a real SP they could still perform well.

Some differences between preinteraction and postinteraction in terms of subthemes were evident under the major theme of learning efficacy. Before the exercise students were focused more on anticipated or previous experiences in using LLMs for personalized learning materials, for example, developing mnemonics, practice questions, or flash cards. This reflects the experience of other medical students [37]. Responses following the exercise were focused on diagnostic patient interaction skills. This is to be expected as students now had actual experience of ChatGPT in this role and knew that this was to be the focus of our study.

Verisimilitude was a major theme in both preinteraction and postinteraction responses. All students mentioned this as a limiting factor. Absence of facial cues, changes in tone, or body language and an inability to develop rapport were all seen as drawbacks of the virtual SP. Some students also mentioned that this impacted their role as physician. For example, a student physician leaning into the patient to show interest, or other types of body language, was redundant in the exercise. Other studies have also highlighted that the output from ChatGPT cannot replicate the true stimuli a physician relies on in a patient visit [28,31,38,39]. We note that virtual patients are developing rapidly, so issues with verisimilitude may be overcome in future, although it may take some time before ChatGPT, specifically, is able to incorporate a visual or physical layer.

Trust as a theme was evident in preinterview responses but had disappeared in postinterview responses. We note that our faculty team, consisting of clinicians and PhD-qualified members, did not notice any “hallucinations” in output, despite multiple repetitions of the exercise. Yanagita et al [40] recently found that high-quality illness scripts, used for improving medical student’s clinical reasoning, could be generated by ChatGPT with relatively few errors. Magalhaes et al [25] also found that a majority of students trusted ChatGPT’s output. Nevertheless, even a single error in ChatGPT output, given multiple health care providers may receive the same output, could affect many patients. It is therefore imperative that the veracity of AI output be thoroughly tested before it is fully integrated into health care and medical education settings [28].

Other subthemes for learning efficacy evident postinteraction were feedback and information overload. Our prompt included a direction for ChatGPT to provide feedback on how students could improve their performance. We note that it was necessary to revise the prompt several times during the study, as initially it provided only positive feedback, which did not help in identifying areas for improvement. Responses under the information overload subtheme suggested that students found that the LLM tended to provide more information in regard to a given question than perhaps a real patient or SP would. This presumably related to the depth of medical knowledge of the LLM but should be considered in further iterations of this exercise. It may be possible to refine the prompt to reduce this effect.

Table 4 compares various platforms able to be used as SPs in medical education, highlighting strengths and limitations across technology, learning efficacy, verisimilitude, and cost. ChatGPT and Claude AI offer affordable, flexible options for unlimited practice but face challenges with uncurated outputs and limited realism in visual and tonal cues. In contrast, platforms such as Body Interact and Soma Lab provide curated cases and interactive features, although often at a higher cost. These findings reinforce that while ChatGPT is a valuable and accessible tool for augmenting SP training, it cannot fully replicate the nuances of human SPs. Addressing limitations such as effective prompt engineering and enhancing realism through improved visual and auditory features could significantly improve its use.

It is possible that the use of ChatGPT as a virtual SP may influence trainees’ sensitivity toward patients through the absence of the genuine human interaction students may have with SPs and real patients [41]. The rapid evolution of AI technologies is addressing these gaps to an extent. For instance, the advanced voice mode (AVM) in newer versions of ChatGPT incorporates natural speech patterns and emotional intonations, which may help simulate more realistic interactions. While AI cannot yet replicate the full nuances of real patient encounters, it serves as a consistent and flexible supplementary tool for medical training. Future advancements in AI capabilities may further enhance their ability to foster empathy and connection, thereby reducing potential concerns around decreased sensitivity in trainees.

A number of recent studies have confirmed the use of ChatGPT, or similar LLMs, as virtual SPs [28,29,42]. Similarly to our study, these studies have highlighted ChatGPT’s potential to reduce resource constraints and improve accessibility in medical training while offering immersive, flexible practice opportunities. At the same time, limitations created by a lack of verisimilitude were also noted.

Both the necessity and challenges of integrating AI, including LLMs, into medical curricula have also been widely acknowledged [43-47]. Addressing inequities in AI models derived from biased training data is crucial, as these can perpetuate disparities in patient care. Strategies to ensure fairness and equitable outcomes, such as transparency in algorithmic design, have been emphasized in recent studies [45,48]. Additionally, resource allocation, faculty training, and the development of tailored content for medical applications add layers of complexity to curricula integration [46,48]. To move forward, curricula must incorporate foundational AI competencies, including ethical considerations, algorithmic fairness, and practical skills such as prompt engineering. Embedding these competencies into existing core courses, rather than as electives, will ensure comprehensive and equitable learning opportunities [43,44,46,48].

To effectively integrate AI into medical curricula, assessments should be designed to balance the use of AI tools while maintaining the integrity of evaluation processes [44]. Educators should implement secure examination protocols, such as locked-down computers and stricter proctoring, to prevent misuse of AI during assessments. However, assessments can also creatively incorporate AI by engaging students in critiquing AI-generated responses or using these tools to identify knowledge gaps and provide tailored feedback. Generative AI can enhance formative assessments by offering immediate and individualized feedback, guiding students’ learning trajectories. We note that our results demonstrate the efficacy of this approach, with the virtual SP providing valuable insights to each student individually on how to improve their patient interactions.

### Study Limitations

The small sample size, comprising only 9 participants from a single institution, and potential ascertainment bias, with tech-savvy volunteers possibly skewing results, limited the study’s generalizability. This lack of diversity in the sample highlights the need for future studies to include larger and more diverse participant pools to enhance the robustness and generalizability of the findings. Our team is currently working on a multicenter, randomized controlled trial with a mixed methods approach. The study uses a convergent parallel mixed methods design and will span 8 months across multiple medical schools. It will use the new AVM of ChatGPT to simulate an SP. The AVM offers several advantages over the original voice mode, including reduced latency and an ability to inject emotion into its voice [29]. The study aims to draw conclusions based on robust statistical data comparing the average percentage improvement of the experimental group with the control groups on Observed Structured Clinical Examination scores, as well as qualitative data exploring students’ learning and perceptions of the AI through focus groups.

### Conclusions

This study found ChatGPT to be an effective supplement, although not a full replacement, to traditional SPs. Students and faculty appreciated its potential, noting benefits such as flexible practice times, reduced stress, and improved diagnostic skills. Some shortcomings were noted, including the need for effective prompt engineering and the lack of nonverbal cues affecting realism. Despite these challenges, its reliability and convenience make it a valuable training tool.

Students’ diagnostic skills were not formally assessed in this study. However, based on their self-reported perceptions and observations of their interactions with ChatGPT, it appears that the AI can be a valuable tool for practicing clinical reasoning and problem-solving skills. Future research could explore the impact of ChatGPT on students’ diagnostic accuracy and clinical performance.

Overall, ChatGPT offers a significant adjunct to traditional SPs, providing accessible, flexible practice opportunities for medical students. The study underscores the importance of integrating prompt engineering into medical curricula and refining AI interactions for balanced information delivery. Continuous advancements in virtual patient technology and AI capabilities, including improved verbal and auditory flow, are expected to further enhance ChatGPT’s use in medical education. Future studies are planned with a larger sample size and using the recently released ChatGPT version 4.o1 with AVM.

## Supplementary material

10.2196/63353 Multimedia Appendix 1 Interview questions.

## Abbreviations

AI: artificial intelligence
AUA: American University of Antigua
AVM: advanced voice mode
LLM: large language model
MUA: Medical University of the Americas
SP: standardized patient
