# Supplementary material for: Assessing ChatGPT’s Capability as a New Age Standardized Patient: Qualitative Study
Source: JMIR Med Educ. 2025 May 20;11:e63353. doi: 10.2196/63353 (PMC12111480; doi:10.2196/63353)
Supplement: Multimedia Appendix 1 [file mededu-v11-e63353-s001.docx]

| **Multimedia Appendix 1: Interview Questions:** **Pre-session Interview Questions:**  1. Background and Experience:  a. How familiar are you with AI-driven tools, particularly in medical education?  b. What previous experiences have you had with simulated or standardized patients in your medical training?  2. Expectations:  a. What are your initial expectations about interacting with a ChatGPT-based simulated patient?  b. What do you think could be the potential benefits or advantages of using ChatGPT-based simulated patients compared to traditional standardized patients?  c. Do you have any concerns or potential challenges you think you might face when interacting with a ChatGPT-based simulated patient?  3. Skills Development:  a. How do you think the ChatGPT-based simulated patient interaction might impact your clinical decision-making and communication skills development?  b. In what ways do you think the ChatGPT-based simulated patient could potentially enhance or hinder your learning experience?  **Post-session Interview Questions:**  1. Experience and Comparison:  a. How did your experience with the ChatGPT-based simulated patient align with your initial expectations?  b. How would you describe the differences between the ChatGPT-based simulated patient interaction and traditional standardized patient interactions, in terms of your learning experience?  2. Benefits and Challenges:  a. What aspects of the ChatGPT-based simulated patient interaction did you find most beneficial or effective for your learning?  b. Were there any challenges or limitations you experienced during the ChatGPT-based simulated patient interaction? If so, how did they impact your learning experience?  3. Skills Development:  a. How do you think the ChatGPT-based simulated patient affected your clinical decision-making and communication skills development?  4. Future Use and Recommendations:  a. Based on your experience, do you think the ChatGPT-based simulated patient could be a valuable supplement or alternative to traditional standardized patient training? Why or why not?  b. What improvements or modifications would you suggest to enhance the effectiveness of ChatGPT-based simulated patients in medical education?  c. Would you recommend incorporating ChatGPT-based simulated patients into medical school training? Why or why not? |
| --- |
